# Screening for Unstable Housing in a Healthcare Setting

**DOI:** 10.3389/phrs.2023.1606438

**Published:** 2023-12-27

**Authors:** Raeann Ng, Nilakshi Gunatillaka, Helen Skouteris, David Blane, Claire Blewitt, Suzanne Nielsen, Elizabeth Sturgiss

**Affiliations:** ^1^ School of Primary and Allied Health Care, Faculty of Medicine, Nursing and Health Sciences, Monash University, Frankston, VIC, Australia; ^2^ Health and Social Care Unit, School of Public Health and Preventive Medicine, Faculty of Medicine, Nursing and Health Sciences, Monash University, Melbourne, VIC, Australia; ^3^ Department of General Practice and Primary Care, Institute of Health and Wellbeing, College of Medical, Veterinary and Life Sciences, University of Glasgow, Glasgow, United Kingdom; ^4^ Monash Addiction Research Centre, Faculty of Medicine, Nursing and Health Sciences, Monash University, Melbourne, VIC, Australia

**Keywords:** screening, unstable housing, homelessness, healthcare, community health

## Abstract

**Objectives:** To describe existing tools for screening patients for unstable housing in a healthcare setting.

**Methods:** A literature search was completed to retrieve articles published in the last 10 years on screening patients for unstable housing in a healthcare setting.

**Results:** The current literature on screening patients for homelessness in healthcare settings describes a variety of tools administered by a range of healthcare providers, but all are based in the United States.

**Conclusion:** The studies revealed the potential for effective screening in healthcare settings and positive engagement of patients and providers with screening. Key areas for future research include innovative methods of screening and evaluation of reliability and validity for a broader range of tools.

## Introduction

Housing, with its physical, psychological and social consequences, is one of the core social determinants of health [1]. The Institute of Global Homelessness (IGH) defines “homelessness” as “living in severely inadequate housing due to a lack of access to minimally adequate housing” [2]. The strong association of unstable housing, poor quality housing and homelessness with poor health outcomes is robustly evidenced in the literature [3].

Healthcare organizations and researchers have approached the development, validation and implementation of screening and addressing housing instability in a variety of ways [3]. There has yet to be a consensus on an accepted definition for housing-related social risks, nor a gold standard screening tool [3]. Effective screening is a crucial first step for the successful implementation of any social care interventions to enhance a holistic approach to patient care [4, 5]. A holistic approach is particularly pertinent in a primary care context, where longer-term patient-clinician relationships allow deeper exploration of various factors affecting an individual’s health. As the patient’s first and ongoing point of contact with the healthcare system, primary care clinicians could facilitate early identification of unstable housing and subsequently provide adequate follow-up to ensure housing issues are addressed. Any screening for unstable housing in a healthcare setting will require social and health system integration and clinician support.

The aim of this narrative review was to synthesize what is known about current screening tools for homelessness that are being used in healthcare settings. More specifically, this review explored the characteristics, administration, utility and impact of these screening tools. The findings of this review will be used to inform the development of an approach for screening in primary care and community settings.

## Methods

We searched PubMed, EBSCOhost and Google Scholar for articles from 2012 to 2022 on screening patients for unstable housing in a community health setting (Supplementary File S1). We focused on the last decade to ensure screening processes are relevant to the current sociocultural context and healthcare infrastructure. We included studies in all healthcare settings. We will use implications for screening in primary care/community health to inform our ongoing research.

## Results

### What Screening Tools for Homelessness Are Currently Used in Healthcare Settings?

#### Settings

We identified 31 relevant screening tools from the 22 studies included in this narrative literature review. All of the included studies were from the United States (US) [3, 4, 6–25]. Healthcare settings included Veterans Health Administration (VHA) outpatient clinics [4, 6, 8, 10–12, 19–21], primary care clinics [3, 9, 13–15, 22, 23, 25], emergency departments [3, 9, 13, 25], specialty outpatient clinics [15, 24, 25], inpatient hospital care [15], home-visiting programs [25], community health centers [18, 25], and veteran homeless center programs [17]. The organizations involved were serving financially disadvantaged patients [3, 13, 18, 23], pediatric patients [22, 25] and rural patients [16]. A significant proportion of studies (*n* = 10) targeted the US veteran population [4, 6, 8, 10–12, 17, 19–21]. This reflects the US Department of Veterans Affairs’ (VA) recognition of this population’s vulnerability to homelessness, and subsequent screening and intervention initiatives [4].

#### Tool Characteristics

All screening tools assessed the patient’s current housing status and/or concerns about future housing instability (Table 1). Seven screening tools utilised direct questioning about housing stability, with questions such as “Over the past year, were you homeless or living in a shelter at any time?” [3], or “Are you worried that in the next 2 months, you may not have stable housing?” [15]. These types of questions relied on the patient’s evaluation of their current living situation. Most screening tools (*n* = 23) included open-ended questions such as, “What is your housing situation today?” [3, 9, 13, 15, 18, 23], “In the last 12 months, how many times have you or your family moved from one home to another?” [15, 25], or “Do you have any concerns about being evicted or not being able to pay the rent?” [15].

**TABLE 1:** Characteristics of the 31 screening tools for unstable housing in a healthcare setting (see Supplementary File S2 for detailed review of each study) (United States, 2013–2022).

Screening tool	Questions related to housing [total questions in survey]	Population studied (patient type, setting)	Provider administering the tool
Veterans Health Administration (VHA) Homelessness Screening Clinical Reminder (HSCR) [4, 6, 7, 10–12, 19, 21]	4 [4]	Veterans, primary care [4, 6, 10–12, 19, 21]	Not specified [4, 6, 10, 19], Physicians [11, 12, 21], Behavioral Health or Social Service Providers [12, 21], Nurses [11, 12, 21], Physician Assistants [12, 21], Advanced Practice Nurses [11, 12], Nurse Practitioners [11, 21], Multiple/other [21]
Accountable Health Communities (AHC) Health-Related Social Needs (HRSN) Screening Tool [3, 9, 13–15, 18, 23]	2 [26]	Adults/children and their caregivers [3, 9, 13]	Not specified
		Publicly insured, primary care [14, 15]	
		Low-income/uninsured, primary care [18]	
		Uninsured/underinsured [23]	
Children’s Health Watch (CHW) Housing Stability Vital Sign Tool [3]	3 [3]	Adults/children and their caregivers	Not specified
Automated Retrieval Console v2.0 (ARC) [8]	N/A	Veterans, primary care	N/A
Your Current Life Situation (YCLS) [14, 15, 18]	4 [9]	All ages, primary care [14, 15]	Not specified
		Low-income/uninsured, primary care [18]	
Structural Vulnerability Assessment Tool [14, 15]	2 [9]	Adults, tertiary care	Not specified
Health Leads [14, 15]	3 [10]	All ages, primary care	Not specified
Protocol for Responding to and Assessing Patient Assets, Risks, and Experiences (PRAPARE) [14, 15]	3 [21]	Adults, primary/secondary care	Not specified
HealthBegins [14, 15, 25]	4 [28]	All ages, primary care [14, 15]	Not specified
		Children and their caregivers, primary care [25]	
HelpSteps (The Online Advocate) [14, 15, 22]	Not specified	Children/young adults, tertiary care [14, 15]	Not specified
		Children and their caregivers, secondary care [22]	
Medical-Legal Partnership (MLP) [14, 15]	2 [10]	Children and their caregivers, secondary care	Not specified
Institute of Medicine (IOM) [14, 15]	1 [24]	Adults, primary care	Not specified
Medicare Total Health Assessment [14, 15]	1 [39]	Publicly insured, primary care	Not specified
Well Rx [14, 15, 22]	1 [11]	Primary care [14, 15]	Not specified
		Children and their caregivers, primary care [22]	
Social History Template [14, 15, 22, 25]	2 [8]	All ages, primary/secondary care [14, 15]	Not specified
		Children and their caregivers, secondary care [22, 25]	
Legal Checkup [14, 15]	1 [1]	Children and their caregivers, secondary care	Not specified
Income, Housing, Education, Legal status, Literacy, Personal Safety (IHELLP) Questionnaire [14, 15, 22, 25]	1–4 [14–23]	Children, their caregivers and others, secondary/tertiary care	Not specified
WE CARE [14, 15, 22, 25]	1 [6]	Children and their caregivers/low-income, primary/secondary care	Not specified
Housing Insecurity Screening Tool [16]	3 [3]	Children and their caregivers	Pediatricians, Nurse Practitioner, Social Worker, Child Psychologist, Registered Nurse (RN), Medical Assistant (MA)
Pilot Homelessness Risk Screener [20]	37 [37]	Veterans, primary care	Social workers
Health-related socioeconomic risk factors (HRSRs) screening survey [24]	Not specified	Adults, secondary care	Not specified
Universal Homeless Housing Screening (UHHS) [17]	Not specified	Veterans, primary care	VANTHCS (Veterans Affairs North Texas Healthcare System) CHCP (Comprehensive Homeless Center Programs) staff members
DeJong et al., 2016 Screening Tool [22]	4 [17]	Children and their caregivers, secondary care	Not specified
Child Health Improvement through Computer Automation-Medical Legal Partnership (CHICA-MLP) process [22]	Not specified	Children and their caregivers, secondary care	Not specified
iScreen [22, 25]	2 [46]	Children and their caregivers, primary/tertiary care	Not specified
Social History Form Embedded in Clinic Electronic Medical Record (EMR) [22, 25]	1 [12]	Children and their caregivers, secondary care	Not specified
Medical-legal advocacy screening questionnaire (MASQ) [22]	Not specified	Children and their caregivers, secondary care	Not specified
Family Map Inventories (FMI) [25]	1 [11]	Children and their families/at-risk families, primary care	Not specified
ASK Tool [25]	1 [13]	Children and their families/low-income, secondary care	Not specified
FAMNEEDS [25]	3 [29]	Children and their families, secondary care	Not specified
Health-Related Social Problems Screener [25]	Not specified	Children and their families/low-income, secondary care	Not specified

AHC, Accountable Health Communities; ARC, Automated Retrieval Console; CHCP, Comprehensive Homeless Center Programs; CHICA-MLP, Child Health Improvement through Computer Automation-Medical Legal Partnership; CHW, Children’s Health Watch; EHR, Electronic health records; FMI, Family Map Inventories; HRSN, Health-Related Social Needs; HRSRs, Health-related socioeconomic risk factors; HSCR, Homelessness Screening Clinical Reminder; IHELLP, Income, Housing, Education, Legal status, Literacy, Personal Safety; IOM, Institute of Medicine; MASQ, Medical-legal advocacy screening questionnaire; MLP, Medical-Legal Partnership; PRAPARE, Protocol for Responding to and Assessing Patient Assets, Risks, and Experiences; UHHS, Universal Homeless Housing Screening; VANTHCS, Veterans Affairs North Texas Health Care System; VHA, Veterans Health Administration; YCLS, Your Current Life Situation.

Screening tools used timeframes ranging from the past 12 months to the upcoming 2 months. Questions were binary (yes/no) [3, 4, 6, 9–16, 18–23, 25], single-answer multiple choice [3, 4, 6, 10–12, 14, 15, 18–22, 25], multiple-answer multiple choice questions [3, 14, 15, 18, 22, 25] or numerical questions [3, 9, 13–15, 18, 20, 23, 25]. Mode of screening administration, in the descending order of number of tools that utilised the particular mode, included self-administration on paper [14–16, 22–25], assisted completion via face-to-face interview between provider and patient (based on a paper form/template in the electronic health record) [4, 6, 10–12, 14, 15, 17, 19–21], self-administration electronically (computer/tablet) [3, 9, 13–15, 18, 22–25] or assisted completion via telephone interview [18, 22, 25]. Some tools had more than one mode of administration. Screening tools often assessed other social risk domains (e.g., food insecurity, financial instability) along with housing-related risks [3, 9, 13–15, 18, 22–25].

Sensitivity and specificity data were only available for the Veterans Health Administration (VHA) Homelessness Screening Clinical Reminder (HSCR) [12, 20]. The HSCR is a screening tool completed via a provider-led interview prompted by the Computerized Patient Record System (CPRS). For the 2017–2018 veteran sample, specificity (86.5%) was significantly greater than sensitivity (32.3%) when screening for homelessness [12]. Similarly, specificity (90.0%) was much higher than sensitivity (16.0%) when screening for risk of homelessness [12]. These statistical values suggest that a considerable proportion of patients experiencing homelessness are not being identified by this screening tool. Several other studies provided data on the living situation of positively screened patients without formal statistical analysis, in order to offer a degree of insight on the tool’s validity [4, 10, 19, 21]. Using the HSCR, over half the patients who screened positive for current homelessness reporting living in a homeless situation for most of the 2 months prior [4, 19].

One of the studies around veterans explored the potential for artificial intelligence to be employed in screening for unstable housing [8]. In this study, an open-source natural language processing (NLP) tool was trained to recognise electronic medical record documents as having either “evidence of homelessness” or “no evidence” [8]. Human review determined a precision of 70% of positive flags for homelessness, suggesting that with further development, NLP tools and other forms of artificial intelligence could be used to create effective and efficient screening processes [8].

#### Characteristics of Providers Who Administer Screening Tools

A wide variety of staff were involved in screening (Table 1). 12 studies did not specify what type of healthcare provider administered the screening tool [3, 4, 6, 9, 10, 13, 15, 18, 19, 22, 23, 25]. In one systematic review, six out of eight tools screening for housing instability had associated training for screening professionals [25]. While there were no studies comparing tool administration between different providers, Montgomery et al. found that veterans screened by a behavioral health or social work provider were almost twice as likely to access services within 30 days after a positive screening than those screened by physicians [21].

#### Acceptability of Screening for Patients and Providers

Six studies discussed the acceptability of the screening tool from the patient and/or provider’s perspective [9, 11, 13, 14, 21, 24]. De Marchis et al. reported that 79% of participants found the Accountable Health Communities’ (AHC) social risk screening tool appropriate and 65% felt comfortable with the inclusion of social risks in electronic health records (EHR) [13]. Pinkerton et al. reported that 92% of patients with ≥1 health-related socioeconomic risk (HRSR) found it appropriate to assess for HRSRs in clinical settings and 72% felt comfortable with EHR documentation [24]. Byhoff et al. conducted interviews with participants who completed a survey that utilised the AHC Screening Tool and found that they viewed screening for social risks as acceptable and crucial [9]. Participants conveyed they wanted their healthcare providers to be informed of their social circumstances, but did not expect them to resolve social issues [9].

Chhabra et al. carried out in-depth interviews with VHA clinical providers about the HSCR which reflected overall positive engagement with the tool [11]. Providers reported that the HSCR encouraged them to integrate patient housing status into routine assessment and subsequent clinical decision-making [11]. A later study in 2021 by Montgomery et al. provided further insight into provider perspectives on the HSCR, reporting that they found the tool easy to administer, but used it as a reminder to enquire about housing rather than following the language of the HSCR verbatim [21]. They attributed this to the overly formal or vague language of the HSCR and concerns about patients’ subjective definitions of what constitutes “stable housing” [21].

From a broader perspective of social risk screening and interventions, Eder et al. reported a majority of the articles (*n* = 31) in their literature search conveying patient satisfaction with and acceptability of screening and interventions, with frequent mentions of improvements in the patient-clinician relationship and high comfort levels [14]. However, 11 articles did raise issues of discomfort and confidentiality which will be further discussed in the “Barriers and Facilitators of Screening” section below [14]. 17 of 18 articles on clinician satisfaction with screening and interventions reported positive findings, including the time-efficiency of screening and subsequent improvements in the patient-clinician relationship, patient care and clinician knowledge and competence [14]. Overall, patient and clinician acceptability of the various screening tools appeared to be high with mutually perceived benefits in the therapeutic relationship and clinical care.

#### Barriers and Facilitators to Screening for Patients and Providers

Six studies [9, 11, 13, 14, 16, 23] discussed potential barriers and/or facilitators to screening for housing instability. De Marchis et al. described that patients were more likely to perceive screening as appropriate if they were in a primary care setting, had previous exposure to healthcare-based social risk screening, or trusted their clinician [13]. Patients were more likely to perceive screening as less appropriate if they had previous experience of healthcare discrimination [13]. Patients were also more comfortable with EHR documentation of social risks if they had previously received assistance with social risks in a healthcare setting [13]. Patients in the qualitative study by Byhoff et al. expressed the importance of a patient-centered approach to social risk screening that shows empathy, compassion and respect [9].

In a quality improvement project, staff reported that they forgot to offer the self-administered screening tool [16]. After the project leader placed the screening tools in a brightly colored folder by their workstation, the screening rate improved from 68% to 45% in Week one and two respectively to 77% in Week three [16]. From interviews with VHA clinical providers about the HSCR, barriers to screening for housing (prior to the HSCR) included providers’ assumptions about who might be at risk, lack of training in medical school and residency, and lack of previous work experience in settings where housing instability is sought to be addressed on a system-wide level [11]. Other potential barriers were raised, such as providers feeling better positioned and more inclined to focus on the medical rather than social factors of the patient’s presenting complaint [11]. This was in consideration of their primarily medical expertise, the time constraints of each brief consultation and lack of familiarity with available resources [11].

Regarding facilitators of screening, incorporation of the HSCR into the Computerized Patient Record System has been effective in prompting screening as clinical providers have to click on it as they proceed through a consultation [11]. Palakshappa et al. found that a major barrier to systematic screening was a lack of personnel, especially in the context of free and charitable clinics as they are often dependent on volunteers [23]. Potential facilitators identified include having a dedicated patient advocate at clinics to assist with social needs [23].

Eder et al. identified patients’ concerns about stigma and privacy, clinician’s concerns about lack of referral resources, and concerns on a health system level about how social risk data is collected and managed across systems [14]. In summary, there is a need for: 1) training for clinicians on screening, 2) time efficiency of the screening tool, 3) incorporation of the screening tool into existing workflow, 4) a robust patient-doctor relationship, and 5) proper integration between healthcare and social care organizations.

### What Action Do Healthcare Providers Take After Screening?

Eight of the 26 publicly available screening tools included a follow-up question to identify if the patient would like to receive assistance for any of the social risks they reported or a prompt for the provider to make an appropriate referral plan [4, 6, 10–12, 15, 17–19, 21, 22, 25]. Seven studies provided data on follow-up post-screening [4, 7, 10, 12, 17, 23, 25]. Four of these studies addressed VA’s HSCR and reported that, of the veterans who screened positive for homelessness or at-risk, at least one in two accepted referrals for assistance or received follow-up services [4, 7, 10, 12]. In another study of the veteran population using the Universal Homeless Housing Screening (UHHS) process, 76% of veterans who had completed the screening were housed within 3 months after the reviewed period, with 60% housed in a UHHS-associated housing program [17].

In a systematic review of screening children (based on parent/caregiver-reported information) for social determinants of health, 10 out of 12 studies (nine different tools) that screen for housing instability reported follow-up procedures post-screening (e.g., providing handouts, referral to community resources, assistance with accessing services) [25]. In the study focusing on social risk screening in free and charitable clinics, most patients were either directly connected to resources (26.5%) or provided with information (61.2%) after a positive screen for housing instability [23]. Overall, there appeared to be follow-up in most cases of screening, although the degree of assistance varies widely from a pamphlet through to being housed.

## Conclusions

Unstable housing is a major problem worldwide, with approximately 150 million people identified as homeless [26] and 1.8 billion without adequate housing [27]. Although global estimates lack precision [28], these figures represent significant proportions of the world’s population and allude to the pervasive impact of unstable housing across countries. While the issue of unstable housing is ubiquitous, it affects each country in different ways depending on the socioeconomic, cultural, structural and political factors [28]. Likewise, any approach to screening for unstable housing in a healthcare setting would vary between different healthcare settings within a country, and even more so between different countries.

Despite the differences between health systems, the insights from existing literature can be drawn on to inform further research and development of screening and intervention processes across various countries. Potential insights include a more nuanced understanding of the multi-faceted and complex nature of unstable housing, and a better appreciation of the practical aspects of screening initiatives and interventions.

The current literature on screening patients for homelessness in healthcare settings describes a variety of tools administered by a range of healthcare providers, but all are based in the United States. We acknowledge the absence of relevant studies from other developed countries as a limitation which reflects the lack of similar screening tools outside of the United States in this publication period. The included tools were similar in the types of questions asked (recent housing status, concerns and indicators of housing instability) and modes of administration (more commonly self-administered rather than provider-led). Overall, patients and providers found screening acceptable, and perceived it as an important part of clinical care. However, there is a lack of data on reliability and validity for some tools, which could be an area for future study. Given advances in technology in healthcare settings, innovative ways to screening could also be explored. There is a lack of data from different countries other than the United States on screening for unstable housing in healthcare settings, highlighting an area requiring further evaluation in the global context.

## Abbreviations

AHC, Accountable Health Communities; ARC, Automated Retrieval Console; CHICA-MLP, Child Health Improvement through Computer Automation-Medical Legal Partnership; CHW, Children’s Health Watch; EHR, Electronic health records; FMI, Family Map Inventories; HRSN, Health-Related Social Needs; HRSR, Health-related socioeconomic risk; HRSRs, Health-related socioeconomic risk factors; HSCR, Homelessness Screening Clinical Reminder; IGH, Institute of Global Homelessness; IHELLP, Income, Housing, Education, Legal status, Literacy, Personal Safety; IOM, Institute of Medicine; MASQ, Medical-legal advocacy screening questionnaire; MLP, Medical-Legal Partnership; NLP, Natural language processing; PRAPARE, Protocol for Responding to and Assessing Patient Assets, Risks, and Experiences; UHHS, Universal Homeless Housing Screening; US, United States; VA, Department of Veterans Affairs; VHA, Veterans Health Administration; YCLS, Your Current Life Situation.
