# Supplementary material for: Screening for Unstable Housing in a Healthcare Setting
Source: Public Health Rev. 2023 Dec 27;44:1606438. doi: 10.3389/phrs.2023.1606438 (PMC10777743; doi:10.3389/phrs.2023.1606438)
Supplement: Supplementary file 1 [file DataSheet2.docx]

**Supplementary File 2: Supplementary Table - Detailed Review of Studies Included in the Narrative Review (‘Screening for Unstable Housing in a Healthcare Setting’) (United States, 2013-2022)**

| First author (Year) | Country | Setting | Population | Intervention (and brief description) | Key Findings |
| --- | --- | --- | --- | --- | --- |
| Quantitative Descriptive Studies | | | | | |
| Byrne et al. (2015) | United States | Veterans Health Administration (VHA) outpatient clinics (Primary care, Mental health, Substance abuse, Other) | **Patient population:** Veterans  **Provider population:**  *Not specified*  *The VHA is run by the Department of Veterans Affairs (VA) and is the largest integrated health care system in the United States, providing care to over 9 million veterans enrolled in the VA health care program (U.S Department of Veterans Affairs, 2022).* | [VHA’s Homelessness Screening Clinical Reminder (HSCR)](https://drive.google.com/file/d/1ZRjuUv8jbuO9idMo49rNJGhfBNR4jqFK/view?usp=sharing)  **Length/content:** 2 primary questions about current homelessness and imminent risk. If yes to any of the questions, patients are asked a further 2 questions about where they have lived for most of the previous two months and if they would like a referral to discuss their living situation further.  **Mode:** This has been launched in the Computerized Patient Record System (CPRS) to prompt a discussion of housing status between the veteran and provider. Administered semi-annually (veterans who screen positive/decline screening) or annually (veterans who screen negative) to veterans accessing VHA outpatient care (without current homeless assistance or residence in a long-term care facility). | **Screening results:**   - More than 4.3 million veterans responded to the HSCR from October 1, 2012 to September 30, 2013 - 0.8% screened positive for current homelessness - 1.0% screened positive for imminent risk of homelessness - Of those who initially screened positive for either homelessness or risk of homelessness and who completed a second screen during the study period, 85.0% resolved their housing instability prior to their second screen (at least six months after the initial screen, during fiscal year 2013)   **Validity:**   - Of the 2499 veterans who screened positive for homelessness or imminent risk of homelessness on both the initial and follow-up screen, 56.9% were living in a homeless situation |
| Bachhuber et al. (2015)  *(Unable to obtain full-text, data extracted from abstract)* | United States | Addiction treatment programs operated by the US Department of Veterans Affairs (VA) | **Patient population:** Veterans initiating treatment with methadone or buprenorphine for opioid use disorder  **Provider population:**  *Not specified* | [VHA’s Homelessness Screening Clinical Reminder (HSCR)](https://drive.google.com/file/d/1ZRjuUv8jbuO9idMo49rNJGhfBNR4jqFK/view?usp=sharing)  (See Byrne et al. (2015)) | **Screening results:**   - 2699 veterans administered the HSCR between October 1, 2013 and September 30, 2014 - 10.2% screened positive for homelessness – approximately 10 times that of the general veteran population accessing care at VA - 5.3% screened positive for risk of homelessness     **Acceptability/Validity:**   - *No data provided* |
| Cusack et al. (2019) | United States | Veterans Health Administration (VHA) outpatient clinics | **Patient population:** Veterans (who had responded to the HSCR in the 90 days prior to presenting for Supportive Services for Veterans Families (SSVF))  **Provider population:**  Physicians, Behavioural Health or Social Service Providers, Nurses, Physician Assistants, Advanced Practice Nurses | [VHA’s Homelessness Screening Clinical Reminder (HSCR)](https://drive.google.com/file/d/1ZRjuUv8jbuO9idMo49rNJGhfBNR4jqFK/view?usp=sharing)  (See Byrne et al. (2015))  *Expected flow through the system: screening (HSCR positive screen) 🡪 triage (VA homeless outreach) 🡪 Referral (SSVF)* | **Screening results:**   - Of the 134 veterans screened between October 2017 to April 2018, 21.6% screened positive for homelessness and 9.7% screened positive for risk of homelessness - Behavioural health and social service providers more frequently documented positive screens, while physicians, nurses and physician assistants more frequently documented negative screens   **Validity:**   - Majority screened negative for housing instability (68.7%) despite presenting for SSVF services within the following 90 days - According to SSVF data - 74.1% of veterans who screened positive for homelessness on the HSCR did not have a home of their own for most of the past 90 days - 61.5% of veterans who screened positive for risk of homelessness did not have a home of their own for most of the past 90 days - HSCR screen for homelessness:   *Sensitivity*: 32.3%  *Specificity*: 86.5%  *Positive predictive value*: 74.1%  *Negative predictive valu*e: 48.3%   - HSCR screen for risk of homelessness:   *Sensitivity*: 16.0%  *Specificity:* 90.0%  *Positive predictive value*: 61.5%  *Negative predictive value*: 48.3% |
| De Marchis et al. (2021) | United States | 7 primary care clinics and 4 emergency departments serving ≥ 30% publicly insured or uninsured patients across 9 states | **Patient population:** Adult patients and adult caregivers of paediatric patients  **Provider population:**  *Not specified* | [Study survey tool](https://drive.google.com/file/d/1UfHcEd_koRm0EVcpPUKbFVsw_G-jyt1z/view?usp=sharing)  **Length/content:** 32-item survey, including 2 questions from the Accountable Health Communities (AHC) Screening Tool and 3 questions from the Children’s HealthWatch (CHW) Housing Stability Vital Sign Tool to assess housing-related risks  **Mode:** Self-completed via electronic tablet  [AHC Health-Related Social Needs (HRSN) Screening Tool](https://drive.google.com/file/d/1oRlrw9o8dpzGUdyCCg1lWBcwPQpySE5p/view?usp=sharing)  10 primary and 16 supplementary questions screening for housing stability, food insecurity, transportation access, utilities security and interpersonal violence  [CHW Housing Stability Vital Sign Tool](https://docs.google.com/document/d/1aJODQJQqlYvXbG8JKQLDqWZXKVtQw0UPMPtksW5RTe8/edit?usp=sharing)  3 questions screening for challenges paying rent/mortgage, multiple moves and current or recent homelessness | **Screening results:**   - Of 835 participants screened between July 2018 to February 2019, 52% screened positive for ≥ 1 housing-related risk question - 32.8% screened discordant: 11.9% screened positive by CHW questions but negative by the AHC tool, and 21% screened positive by the AHC tool but negative by CHW - Only 18.7% of all participants screened positive by both tools - Results regarding homelessness/housing instability - 17% positive for current/anticipated housing instability (AHC) - 23% positive for difficulty paying rent/mortgage in past 12 months (CHW) - 10% reporting ≥ 2 moves in past 12 months (CHW) - 8% positive for experience of homelessness currently/previous 12 months (CHW)   **Acceptability/Validity**:   - *No data provided* |
| De Marchis et al. (2019) | United States | 6 primary care clinics and 4 emergency departments serving ≥ 30% publicly insured or uninsured patients across 9 states | **Patient population:** Adult patients and adult caregivers of paediatric patients  **Provider population:**  Not specified | [Accountable Health Communities (AHC) Health-Related Social Needs (HRSN) Screening Tool](https://drive.google.com/file/d/1oRlrw9o8dpzGUdyCCg1lWBcwPQpySE5p/view?usp=sharing)  **Length/content:** (See De Marchis et al. (2021))  **Mode:** Self-completed via a tablet device  *(22 Questions about appropriateness of screening and comfort with including social risk data in electronic health records were included in the survey as well)* | **Screening results:**   - Among the 969 participants screened from July 2018 to February 2019, 61.4% screened positive for at least 1 of 5 social risks based on AHC cut points on the screening instrument   **Acceptability:**   - 88.4% did not skip or select *I prefer not to answer* for any of the 10 AHC questions - Screening was reported as appropriate by 79% of participants, 14% were neutral - 65% reported comfort including social risks in electronic health records, 17% were neutral - In adjusted models, higher perceived screening appropriateness was associated with previous exposure to healthcare-based social risk screening, trust in clinicians, and recruitment from a primary care setting - Lower perceived screening appropriateness was associated with previous experience of healthcare discrimination - Higher comfort with electronic health record documentation was associated with previously receiving assistance with social risks in a healthcare setting |
| Fargo et al. (2017) | United States | Veterans Health Administration (VHA) outpatient clinics | **Patient population:** Veterans  **Provider population:**  Not specified | [VHA’s Homelessness Screening Clinical Reminder (HSCR)](https://drive.google.com/file/d/1ZRjuUv8jbuO9idMo49rNJGhfBNR4jqFK/view?usp=sharing)  (See Byrne et al. (2015)) | **Screening results:**   - Of 5,771,496 veterans screened from October 1, 2012 to September 20, 2014, 0.8% screened positive for current housing instability and 1.0% were positive for risk of housing instability   **Validity:**   - Among those who screened positive for housing instability, 61.9% were living in a homeless situation, 24.5% were not - Among those who screened positive for risk of housing instability, 25.1% were living in a homeless situation, 65.3% were not - Administrative evidence for homelessness significantly varied depending on whether veterans accepted or declined a referral for services - 61.3% of those with a positive screen for housing instability who accepted services showed administrative evidence for homelessness as compared to only 19.0% who declined - 31.5% of those with a positive screen for risk of housing instability who accepted services showed administrative evidence for homelessness as compared to only 7.0%% who declined |
| Gundlapalli et al. (2013) | United States | Veterans Health Administration (VHA) outpatient clinics | **Patient population:** Veterans  **Provider population:**  *Not specified* | Automated Retrieval Console v2.0 (ARC) – an open-source natural language processing (NLP) tool that retrieves ‘documents like this one’ based on a training set that contains sufficient numbers of positive and negative classifications.  Using a human-reviewed reference standard corpus of clinical documents of veterans with evidence of homelessness and those without, this tool was trained to classify documents as having either ‘evidence of homelessness’ or ‘no evidence’. | - The best performing model based on document level work-flow performed well on a test set (Precision 94%, Recall 97%, F-Measure 96)*   **Precision – fracture of retrieved documents that are relevant, a proxy for positive predictive value, Recall – a fracture of relevant documents retrieved, a proxy for sensitivity, F-measure – harmonic mean of precision and recall*  **Screening results:**   - Processing of a naïve set of 10000 randomly selected documents from the Veterans’ Affairs (VA) using this model indicated a 4.7% prevalence of homelessness in 2009, with 463 documents flagged as positive   **Validity:**   - Human review noted a precision of 70% of these flags resulting in an adjusted prevalence of homelessness of 3.3%, which matches current VA estimates - This study has shown the potential effectiveness and efficiency of using NLP tools to screen for homelessness from electronic medical records within a reasonable time frame |
| Henrikson et al. (2019) | United States | Primary care, Specialty care and Inpatient settings | **Patient population:** All ages, Medicare/Medicaid patients, Adults, Children/Young adults, Children and Families, Web volunteer registry, Adult women  **Provider population:**  Not specified | 19 of the 21 tools included in this systematic review assessed for homelessness. The tools created after 2000 (n=15) are listed here:   - [Your Current Life Situation](https://drive.google.com/file/d/1qZAd-Eg2hEqG_Wkb7BTa6oaPK7HIYn9H/view?usp=sharing) - [Accountable Health Communities (AHC) Health-Related Social Needs (HRSN) Screening Tool](https://drive.google.com/file/d/1oRlrw9o8dpzGUdyCCg1lWBcwPQpySE5p/view?usp=sharing) - [Structural Vulnerability Assessment Tool](https://drive.google.com/file/d/1OQFesn3iDemZz38rFaF7GhjlTM8P3IKG/view?usp=sharing) - [Health Leads](https://drive.google.com/file/d/1Z4q7bv_5lgus9UMksiesU6qND7Ll1jA4/view?usp=sharing) - [Protocol for Responding to and Assessing Patient Assets, Risks, and Experiences (PRAPARE)](https://drive.google.com/file/d/1RoqA8cPCvyUL-3H8EseWPM07nDWW7oy0/view?usp=sharing) - [HealthBegins](https://drive.google.com/file/d/163AAcaGsPzFkaDisZxmJ0f8s0HJCiwr2/view?usp=sharing) - [HelpSteps (The Online Advocate) – table of survey categories linked](https://docs.google.com/document/d/1nNwbYZY7-I1lbtTOWz2gPT6WcPvKL6ISGYN_HhHbiAM/edit?usp=sharing) - [Medical-Legal Partnership](https://drive.google.com/file/d/1fWfh5GG4G-VlHoFX_x4C7zV_7wWmA0DY/view?usp=sharing) - [Institute of Medicine (IOM)](https://drive.google.com/file/d/17DHalwCeJ8aELfR5XBvepVTW6b_gZ5oE/view?usp=sharing) - [Medicare Total Health Assessment](https://drive.google.com/file/d/1eJmPRgNlC4OxfsWYs1rLZt0dja0zEDWP/view?usp=sharing) - [Well Rx](https://drive.google.com/file/d/181BizcLfiJBE5Gb-7pMZj9XdtNa5z5rb/view?usp=sharing) - [Social History Template](https://docs.google.com/document/d/1wUKs-uqZHTGef6Q-PlB_Bd1DiGtgULw_YF8M3fG_Lvo/edit?usp=sharing) - [Legal Checkup](https://docs.google.com/document/d/1JtxiNNu9AS6Ym-JRBVN4vB2EvwSdPXp6BrWt2Lv5pGY/edit?usp=sharing) - Income, Housing, Education, Legal status, Literacy, Personal Safety (IHELLP) Questionnaire (4 of 5 variations cited able to be obtained): - [Kenyon et al. (2007)](https://docs.google.com/document/d/1fMqEO6w-bmoKDxjuqVmo6YM6rKW5D1V45DwdsV6CnrU/edit?usp=sharing) - [Colvin et al. (2016)](https://drive.google.com/file/d/1ZOIQ4NPJ9by7qAdYC5IPblqRfzxWfDEk/view?usp=sharing) - [Ko et al. (2016)](https://docs.google.com/document/d/1XvTK0q8sxWA_HKt1zECis0zRcCAZ_u6Fg9rbN7snIHM/edit?usp=sharing) - [Patel et al. (2018)](https://docs.google.com/document/d/18TF4Kk2mz5VUpp-vnNihGoZxmr5wPX3VEmfBi9I2ijE/edit?usp=sharing) - [WE CARE](https://drive.google.com/file/d/11Xnw7olX2yH5lczVGduJ8iDg5YKvkn-p/view?usp=sharing) | **See** [**Appendix A**](#_Appendix_A:_Psychometric_2) **for detailed summary tables**  **Tool characteristics:**   - Year created ranges from 1992-2018, 52.4% of included tools were developed in the previous 5 years (from 2018) - Of the 21 tools, 52.4% were used with adults or all-ages populations, whereas 33.3% were used only in paediatric or adolescent populations - 90.5% were used in ambulatory care settings, typically primary care but sometimes specialty care - Number of items varied widely from 7 to 130 - Range of administration time was 3 to 75 minutes for the tools where these data were available - Tools were administered by paper (52.4%), verbally (42.9%), or electronically (33.3%) - Multiple modes of administration were used with 8 tools (38.1%) - No data on method of administration were available for 5 tools   **Quality/Psychometric evidence:**   - None reported following all 8 steps of gold standard measurement in measure development - At least 1 gold standard method was used in development of 85.7% of tools - A clear construct definition was provided for only 9.5% of tools, but working with experts to generate an initial item list was reported for 57.1% - Developers of 42.9% of tools conducted pilot testing using a representative population sample - Reliability/validity testing data were available for 38.1% of tools, but only 9.5% reported results from validity or reliability pilot testing with a representative sample - For each tool, 1 to 10 empirical uses were identified in the published literature - 71.4% had been modified from the original format in subsequent empirical uses - Very little psychometric evidence reported - No data on discriminant validity, known-groups validity, structural validity, or responsiveness were reported on any tool - Tools reporting on internal consistency (14.3%) were rated as adequate (9.5%) or excellent (4.8%), whereas predictive validity (14.3%) scores were all poor (4.8%) or minimal (9.5%) - Psychometric scores on the PAPERS scale ranged from −1 to 9 (mode, 2), placing all included tools in the third lowest quartile of possible scores   **Pragmatic evidence:**   - 95.2% had some pragmatic properties described - Of these, 66.7% were available in the public domain - 70% of tools were rated excellent on accessible language, with 65% written at the 8^th^ grade reading level or below - Data on ease of training were reported for only 19% of tools, but all 4 received adequate, good, or excellent ratings - Ease of interpretation was assessed for only 33.3% of tools, 4 of 7 received a minimal rating - Brevity was assessable for 81% of tools and of those tools 88.2% received good or excellent - Only 23.8% of tools contained 1-10 items - Overall pragmatic scores ranged from −1 to 20 (most pragmatic would be a score of 36) - Of the 21 tools, 11 were in the top 2 quartiles of total possible scores; 3 were in the highest quartile (WeCare, Safe Environment for Every Kid, Survey of Well-Being of Young Children) |
| Higginbotham et al. (2019) | United States | Primary care setting – rural health clinic | ***Patient population:*** *Adult caregivers of young children ≤ age 5 years*  ***Provider population:***  *Paediatricians, Nurse Practitioner, Social Worker, Child Psychologist, Registered Nurse (RN), Medical Assistant (MA)**  **The RN and MA gave the screening tool to the parents/guardians during the check-in process, retrieved and reviewed the screening tool, alerted the providers of positive screens* | Food insecurity (FI) and housing insecurity (HI) self-administered screening tool  **Length/Content:** 5-items including a 2-item food insecurity (FI) tool from the United States Department of Agriculture (USDA) 18-item Household Food Security Survey and a 3-item housing insecurity (HI) screen  **Mode:** Self-administered paper-based survey  3-item HI Screen   - “Within the past 12 months there were two or more people per bedroom?” (Yes/No) - “Within the past 12 months we were temporarily staying with another family or had another family staying with us?” (Yes/No) - “Within the past 12 months we moved more than once?” (Yes/No) | **Screening results:**   - Of the 53 patients/families, 16.9% screened positive for financial instability (FI) - 18.8% screened positive for housing instability (HI) - 11.3% screened positive for both FI and HI   **Acceptability:**   - Staff screening rate for administering the screening tool was 63% overall over 3 weeks - Staff reported that human error, forgetting, was the main reason screening tools were not given to patients - The screening rate improved from 68% and 45% in Week 1 and 2 respectively to 77.4% in Week 3 after the project leader placed blank FI/HI screening tools in a brightly coloured folder by the RN and MA’s workstation |
| Lewis et al. (2020) | United States | Kaiser Permanente Community Health | **Patient population:** Kaiser Permanente Southern California subsidised exchange members – income typically too high for many government and/or community financial assistance programs, but low enough to cause financial strain  **Provider population:**  Not specified | Survey:  **Length/content:** 48 questions, including Your Current Life Situation (YCLS) and Accountable Health Communities’ (AHC) Screening tools and previously validated scales, that took 10-15 min to complete in either English/Spanish  **Mode:** Online or via telephone interview  [YCLS Screening tool](https://drive.google.com/file/d/1qZAd-Eg2hEqG_Wkb7BTa6oaPK7HIYn9H/view?usp=sharing):  9 questions about patient risks over the previous 3 months related to living situation, financial hardship, food insecurity, transportation, support for activities of daily living, stress, and desire for help  [AHC Health-Related Social Needs (HRSN) Screening Tool](https://drive.google.com/file/d/1oRlrw9o8dpzGUdyCCg1lWBcwPQpySE5p/view?usp=sharing)  (See De Marchis et al. (2021)) | **Screening results:**   - Insecure housing was reported by 10% on the AHC screening tool and 4% on the YCLS screening tool - On the AHC tool, the percentage reporting social risks ranged from 7% to 29% - On the YCLS tool the percentage reporting social risks ranged from 4% to 23%   **Validity:**   - Concurrent validity: agreement between the AHC and YCLS tools was substantial on all items (kappas > 0.60) except for housing quality (kappa 0.52) - Kappa 0.79 for insecure housing - Predictive validity: 4 out of 6 screening questions on the AHC tool and 4 out of 7 on the YCLS tool were associated with self-rated health (P < 0.03) - No social risks were associated with flu shot receipt except utilities on the AHC tool (P=0.028) |
| Montgomery et al. (2013) | United States | Veterans Health Administration (VHA) outpatient clinics | **Patient Population:** Veterans  **Provider Population:**  *Not specified* | [VHA’s Homelessness Screening Clinical Reminder (HSCR)](https://drive.google.com/file/d/1ZRjuUv8jbuO9idMo49rNJGhfBNR4jqFK/view?usp=sharing)  (See Byrne et al. (2015)) | **Screening results:**   - Of the 1,398,925 veterans screened between October 1, 2012 and January 10, 2013, 0.9% reported current unstable housing or homelessness - 1.2% reported being at risk for housing instability   **Validity:** *(based on reported living situation for the 2 month period before date of screening)*   - Of the veterans that screened positive for current unstable housing or homelessness, 58.4% were living in a homeless situation (living with friend/family, in a motel/hotel, in a shelter, or on the street) - Of the veterans that screened positive for risk of homelessness, 22.7% were living in a homeless situation |
| Montgomery et al. (2014) | United States | Veterans Health Administration (VHA) social work, mental health, substance use outpatient clinics at 4 locations in Pennsylvania and Delaware | **Patient population:** Veterans  **Provider population:**  Social workers administered the pilot instrument during the field test | [Pilot Homelessness Risk Screener](https://drive.google.com/file/d/1bkdTzZBq5tqkuCDyjxFv5LyVljoFhNLH/view?usp=sharing)  **Length/Content:** 37 questions - Stage I assesses for imminent risk of homelessness, Stage II determines degree/severity of homelessness risk  **Mode:** Social workers administered both stages to each veteran by reading the items and then entering the veteran’s response into his/her electronic medical record, requiring approximately 5 min  *based on initial psychometric analyses of the pilot instrument, items were reduced (primarily due to low positive response rates) and several questions amended to produce the 25-item instrument | **Screening results:**   - Of 397 veterans, 33% screened positive for imminent risk of homelessness from December 2011 to February 2012   **Reliability/Validity:**   - The reliability coefficient for the instrument was 0.85, indicating good internal consistency - Internal consistency reliability of items was 0.61 for Stage I and 0.82 for Stage II - Lower reliability of Stage I portion of instrument largely explained by small no. of binary-scored items (n=2) - Internal consistency reliability of the final instrument was the same as the pilot instrument - Convergent validity: With the exception of four items—living in unsubsidized housing, paying for housing expenses, having friends or family the household could live with, and having a physical disability—a positive response to each of the Stage II items increased the likelihood that a veteran would screen positive for homelessness risk - Sensitivity and specificity were both maximized when the risk severity score* was 6 for Stage I item 1 (0.76 and 0.77, respectively) and item 2 (0.83 and 0.86, respectively), and 5 for the result of Stage I screening (positive or negative) (0.72 and 0.73, respectively).   **Summed risk severity score calculated by adding the weights for Stage II items with a positive response (range from 0 to 32)* |
| Palakshappa et al. (2020) | United States | Clinics part of the North Carolina Association of Free and Charitable Clinics | **Patient population:** Uninsured and underinsured patients in North Carolina  **Provider population:**  Not specified | [Accountable Health Communities (AHC) Health-Related Social Needs (HRSN) Screening Tool](https://drive.google.com/file/d/1oRlrw9o8dpzGUdyCCg1lWBcwPQpySE5p/view?usp=sharing)  (See De Marchis et al. (2021))  A web-based survey was developed to assess current social determinants of health (SDH) screening practices. The SDH domains included in the survey relate to the AHC HRSN Screening Tool. | **Survey results:**   - Of the 55 clinics, 61.8% reported always screening for at least one social determinant of heath (SDOH) - In regard to screening for the item, ‘Does the patient have housing’, 38.2% of clinics reported ‘Always’, 50.9% reported ‘Sometimes’ and 10.9% reported ‘Never’ - In regard to addressing the item, ‘Does the patient have housing’, 26.5% reported ‘Directly connected to resources’, 61.22% reported ‘Provided with information’ and 12.2% reported ‘Other’ |
| Pinkerton et al. (2022) | United States | Outpatient obstetrical and gynaecologic clinics | **Patient population:** Patients aged ≥18 years  **Provider population:**  *Not specified* | Health-related socioeconomic risk factors (HRSRs) screening survey  **Length/content**: Assessment of sociodemographic characteristics, HRSR status, and attitudes toward HRSR screening and documentation in electronic health records (EHRs). Length not specified.  **Mode:** Self-administered survey | **Screening results:**   - Of the 133 patients between April 2019 and June 2019, 47% of patients reported ≥1 HRSR – of these patients 60% desired assistance with HRSRs (32% overall) - 25% of patients reported housing instability - The desire for assistance with HRSRs was endorsed by 65% of patients with housing instability   **Acceptability:**   - Among all patients with ≥1 HRSR, 72% were comfortable with EHR documentation (66% overall), and 92% felt it was appropriate to assess for HRSRs in clinical settings (82% overall) |
| Hooshyar et al. (2015) | United States | VA North Texas Health Care System (VANTHCS) Comprehensive Homeless Center Programs (CHCP) | **Patient population:**  Veterans  **Provider population:**  VANTHCS CHCP staff members | Universal Homeless Housing Screening (UHHS)  **Length/content:** adapted from the Homelessness Screening Clinical Reminder (HSCR) in order to create a standardised process through which veterans are screened for all Comprehensive Homeless Center Programs (CHCP) housing programs during a single screening assessment. This tool consists of the 4 sections: History, Decision Tree, Specific Program Eligibility Criteria and Plan.  **Mode:** These sections exist as templates in the Computerized Patient Record System (CPRS). CHCP staff member performs the screening. | **Screening results:**   - Of 392 veterans screened from August 26, 2013 to November 27, 2013, 95% were eligible for at least one program   **Validity:**   - In regards to residence the night prior to UHHS screening, 64.6% were living in a homeless situation |
| Qualitative Studies | | | | | |
| Byhoff et al. (2019) | United States | 6 primary care clinics and 4 emergency departments across 9 states | **Patient population:** Adult patients and adult caregivers of paediatric patients  **Provider population:**  *Not specified* | [Accountable Health Communities (AHC) Health-Related Social Needs (HRSN) Screening Tool](https://drive.google.com/file/d/1oRlrw9o8dpzGUdyCCg1lWBcwPQpySE5p/view?usp=sharing)  (See De Marchis et al. (2021))    *(Semi-structured interviews were conducted with patients and caregivers who completed a survey that included the AHC Screening Tool between July 2018 and February 2019)* | **Acceptability:**   - Participants believed screening for social risks is important, acceptable and necessary. - Participants expressed insight into the impacts of social risks on both mental and physical health. - Participants emphasised the importance of patient-centred implementation of social risk screening that shows empathy, compassion and respect. - Participants recognized limits to the healthcare sector’s capacity to address or resolve social risks – they wanted their healthcare teams to be aware of social situations but did not expect them to resolve social problems. |
| Chhabra et al. (2019) | United States | Veterans Health Administration (VHA) outpatient clinics (Primary Care, Women’s Health, Geriatrics, Psychiatry, Homeless Patient Aligned Care Teams) | **Patient population:** Veterans  **Provider population:**  Physicians, Nurse Practitioner, Advanced Practice Psychiatric Nurse | [VHA’s Homelessness Screening Clinical Reminder (HSCR)](https://drive.google.com/file/d/1ZRjuUv8jbuO9idMo49rNJGhfBNR4jqFK/view?usp=sharing)  (See Byrne et al. (2015))  *(In-depth semi-structured interviews were carried out with VHA clinical providers)* | **Acceptability:**   - Providers reported that the HSCR prompted them to incorporate patient housing status into routine assessment, which they typically did not do prior to its implementation - Providers discussed adverse impacts of housing instability on patients’ overall health and described how they factored patients’ housing instability into clinical decision-making - Although providers viewed the health system as having an important role in addressing housing concerns, there were mixed opinions on whether it was the role of providers to directly administer the screening. |
| Morone (2017) | United States | Paediatric primary care clinics | **Patient population:** Paediatric populations in the United States  **Provider population:**  *Not specified* | Out of the 13 studies included in the systematic review, there were 10 screening tools assessing for housing insecurity (among other social domains)   - [Social History Template](https://docs.google.com/document/d/1wUKs-uqZHTGef6Q-PlB_Bd1DiGtgULw_YF8M3fG_Lvo/edit?usp=sharing) - [DeJong et al., 2016 Screening Tool](https://journals.sagepub.com/na101/home/literatum/publisher/sage/journals/content/cpja/2016/cpja_55_3/0009922815591959/20160830/images/large/10.1177_0009922815591959-table1.jpeg) - [HelpSteps (The Online Advocate) – table of survey categories linked](https://docs.google.com/document/d/1nNwbYZY7-I1lbtTOWz2gPT6WcPvKL6ISGYN_HhHbiAM/edit?usp=sharing)  – 2 studies created surveys based on this tool - [WE CARE](https://drive.google.com/file/d/11Xnw7olX2yH5lczVGduJ8iDg5YKvkn-p/view?usp=sharing) - Child Health Improvement through Computer Automation-Medical Legal Partnership (CHICA-MLP) process - [iScreen](https://drive.google.com/file/d/174fpz1A5ssrZNY2lCNUxkMKZYhagg0kr/view?usp=sharing) - [Social History Form Embedded in Clinic Electronic Medical Record (EMR)](https://docs.google.com/document/d/1omnDFV7zqJ0Mq-BNZjwppEFkdOwdIYtZt-RUeGKLnbM/edit?usp=sharing) - Medical-legal advocacy screening questionnaire (MASQ) - [IHELLP Mnemonic](https://docs.google.com/document/d/1fMqEO6w-bmoKDxjuqVmo6YM6rKW5D1V45DwdsV6CnrU/edit?usp=sharing) - [Well Rx](https://drive.google.com/file/d/181BizcLfiJBE5Gb-7pMZj9XdtNa5z5rb/view?usp=sharing) | **See** [**Appendix B**](#_Appendix_B:_An) **for a detailed summary table**  **Tool characteristics:**   - Economic Stability (poverty, employment, food insecurity, housing instability) was the primary and most comprehensive SDOH domain assessed across all 13 studies - Predominant administration methods used to assess the SDOH included web-based and paper and pencil based self-report questionnaires   **Validity:**   - 62% of studies did not use any methods to validate the screening tools and methods they employed - Out of the screening tools assessing for housing insecurity, these have been previously validated: The Online Advocate, WE CARE, iScreen (used sample of questions from previously validated surveys), MASQ (sensitivity and specificity determined)   **Tool development:**   - 77% of studies included a discussion of how their tool was initially developed - Only 30% included community members in the initial development of the screening tool - 38% relied on using a limited sampling of questions from various previously validated single domain SDOH measures and assessment tools |
| Sokol et al. (2019) | United States | Paediatric primary care clinics, Emergency departments, Home-visiting programs, Community health centres, Hospital-based paediatric clinics | **Patient population:** Pediatric patients (age ≤ 18)  **Provider population:**  *Not specified* | 11 Social Determinants of Health (SDOH) screening tools, 9 of these assess for housing instability (among other social domains)   - [iScreen](https://drive.google.com/file/d/174fpz1A5ssrZNY2lCNUxkMKZYhagg0kr/view?usp=sharing) - [HealthBegins](https://drive.google.com/file/d/163AAcaGsPzFkaDisZxmJ0f8s0HJCiwr2/view?usp=sharing) - [Family Map Inventories (FMI)](https://docs.google.com/document/d/1DjgFaWOUzO1OHyu6ey81RKQlVqYuZeNmuYZmZGuysfE/edit?usp=sharing) - [ASK Tool](https://drive.google.com/file/d/1Fu50I5FBaFtRn8qgAoP43nxG8UQ2bYwH/view?usp=sharing) - [IHELP](https://drive.google.com/file/d/1ZOIQ4NPJ9by7qAdYC5IPblqRfzxWfDEk/view?usp=sharing) - [WE CARE survey instrument](https://drive.google.com/file/d/11Xnw7olX2yH5lczVGduJ8iDg5YKvkn-p/view?usp=sharing) - [FAMNEEDS](https://drive.google.com/file/d/1yHFDtVuDwtmTXmBMwSMxETJwDRdOkOg7/view?usp=sharing) - [Social History Template](https://docs.google.com/document/d/1wUKs-uqZHTGef6Q-PlB_Bd1DiGtgULw_YF8M3fG_Lvo/edit?usp=sharing) - Health-Related Social Problems screener | **See** [**Appendix C**](#_Appendix_C:_Screening) **for detailed summary tables**  **Tool characteristics:**   - Screening was conducted in a doctor’s or paediatrician’s office for 6 out of 8 tools - Average time to complete the screener ranged from 4-20 minutes - Mode of administration: Computer or tablet, face-to-face/phone interviews, paper and pencil   **Validity:**   - 2 tools had validity and/or reliability assessed in ≥ 1 study (IHELP, WE CARE) |
| Eder et al. (2021) | United States | Clinical care settings, majority of which are primary care settings | **Patient population:** Patients of all ages  **Provider population:**  *Not specified* | Guiding Question 1 (Social Risk Screening Tools) discussed screening tools identified in a 2019 systematic review (Henrikson et al., 2019) which has been included in this summary table – refer to the relevant row | **Guiding Question 1 (Social Risk Screening Tools):**  For the technical brief, the way in which the 18 tools assess target social risk domains was examined   - 12 tools frame 1 or more questions in terms of “concerns,” “worries,” “problems,” and/or “troubles” to detect patient-identified social needs - Only 5 tools ask whether patients would like help with needs they have identified - Questions regarding housing address current housing status, housing quality, and concerns about future homelessness - Seven tools addressing financial strain ask about ability to cover basic necessities (food, housing, medical care, heat) or “make ends meet”. - Key informants reported selecting screening tools because they were clinically validated, had a limited time burden, would result in nationally comparative data, met organisational needs, or incorporated the most important patient needs   **Guiding Question 4 (Challenges of Social Risk Screening and Interventions):**   - See [Appendix D](#_Appendix_D:_Screening) for detailed summary table - Perceived challenges based on information gathered from key informants and a literature review - Patients’ concerns about stigma and privacy - Clinicians’ concerns about lack of referral resources - Health system level concerns about social risk data collection and management by health care organisation and partnering organisations   **Question 5 (Acceptability and Unintended Consequences of Social Risk Screening and Interventions)**   - 52 studies provided data on patient-or clinician-reported satisfaction or challenges after implementation of social risk screening or interventions - Patients - 31 articles included positive patient reports of satisfaction with and acceptability of screening and interventions, often referring to improvements in the patient-clinician relationship and high comfort levels - 11 articles reported on challenges or unintended consequences of screening or intervention for patients, including discomfort (e.g shame about social risks) and confidentiality issues (e.g fear of legal repercussions such as being reported for child maltreatment due to food insecurity) - Clinicians - 17 articles reported positively on clinician satisfaction with screening and interventions, with clinicians stating that screening was not overly time-consuming and led to improvements in the patient-clinician relationship, patient care, and clinician knowledge and competence - The 1 negative report was related to difficulty in incorporating the intervention into clinician schedules - 15 articles reported on challenges or unintended consequences of social risk screening or interventions for clinicians, including lack of time to conduct screening or follow-up on positive results and inability to track the success of referrals |
| Mixed Methods Studies | | | | | |
| Montgomery et al. (2021) | United States | Veterans Health Administration (VHA) outpatient clinics (Primary care, Behavioural health, Substance use, Other) | **Patient population:** Veterans  **Provider population:**  Physicians, Behavioural Health Providers, Nurse Practitioners, Physician Assistants, Nurses, Multiple/other | [VHA’s Homelessness Screening Clinical Reminder (HSCR)](https://drive.google.com/file/d/1ZRjuUv8jbuO9idMo49rNJGhfBNR4jqFK/view?usp=sharing)  (See Byrne et al. (2015)) | **Quantitative:**   - More than 5.78 million unique veterans responded to the HSCR - 0.84% screened positive for current homelessness and 0.96% screened positive for imminent risk of homelessness - Of the 100,022 veterans who screened positive and did not have any missing data, 42.6% were living in a homeless situation:   **Qualitative:** (collected during in-depth interviews with 22 health care providers and six Homeless Program staff):   - Providers reported that they found the HSCR easy to administer, but described the language as too formal or vague, and reported that they used the HSCR as a reminder to ask about housing rather than adhering to the language of the HSCR verbatim - In particular, providers noted concerns about patients’ subjective definitions of what constitutes “stable housing,” and worried that some patients would not self-identify their situation as unstable when, from the clinician’s perspective, it actually was (e.g., living temporarily with a family member or “couch-surfing”) - As a result, several providers reported supplementing or substituting the HSCR questions with their own questions to assess patients’ housing stability. - These questions were generally shorter and more specific; one physician in a specialty care clinic described asking, “Who are you living with?” while a general primary care physician said he asked his patients “Where are you living?” |

# References:

1. Byrne T, Fargo JD, Montgomery AE, Roberts CB, Culhane DP, Kane V. Screening for Homelessness in the Veterans Health Administration: Monitoring Housing Stability through Repeat Screening. Public Health Rep. 2015;130(6):684-92.

2. U.S Department of Veterans Affairs. Veterans Health Administration United States: U.S Department of Veterans Affairs 2022 [updated May 24 2022; cited 2022 8 July ]. Available from: https://www.va.gov/health/aboutVHA.asp.

3. Bachhuber MA, Roberts CB, Metraux S, Montgomery AE. Screening for homelessness among individuals initiating medication-assisted treatment for opioid use disorder in the Veterans Health Administration. J Opioid Manag. 2015;11(6):459-62.

4. Cusack M, Montgomery AE, Hunt-Johnson N, Dichter M, True G. Making Connections: Understanding How Screening, Triage, and Referral Processes Can Promote Veteran Housing Stability. Soc Work Public Health. 2019;34(6):483-91.

5. De Marchis EH, Ettinger de Cuba SA, Chang L, Sheward RS, Doran KM, Gottlieb LM, et al. Screening Discordance and Characteristics of Patients With Housing-Related Social Risks. Am J Prev Med. 2021;61(1):e1-e12.

6. De Marchis EH, Hessler D, Fichtenberg C, Adler N, Byhoff E, Cohen AJ, et al. Part I: A Quantitative Study of Social Risk Screening Acceptability in Patients and Caregivers. Am J Prev Med. 2019;57(6 Suppl 1):S25-S37.

7. Fargo JD, Montgomery AE, Byrne T, Brignone E, Cusack M, Gundlapalli AV. Needles in a Haystack: Screening and Healthcare System Evidence for Homelessness. Stud Health Technol Inform. 2017;235:574-8.

8. Gundlapalli AV, Carter ME, Palmer M, Ginter T, Redd A, Pickard S, et al. Using natural language processing on the free text of clinical documents to screen for evidence of homelessness among US veterans. AMIA Annu Symp Proc. 2013;2013:537-46.

9. Henrikson NB, Blasi PR, Dorsey CN, Mettert KD, Nguyen MB, Walsh-Bailey C, et al. Psychometric and Pragmatic Properties of Social Risk Screening Tools: A Systematic Review. Am J Prev Med. 2019;57(6 Suppl 1):S13-S24.

10. Higginbotham K, Davis Crutcher T, Karp SM. Screening for Social Determinants of Health at Well-Child Appointments: A Quality Improvement Project. Nursing Clinics of North America. 2019;54(1):141-8.

11. Lewis CC, Wellman R, Jones SMW, Walsh-Bailey C, Thompson E, Derus A, et al. Comparing the performance of two social risk screening tools in a vulnerable subpopulation. J Family Med Prim Care. 2020;9(9):5026-34.

12. Montgomery AE, Fargo JD, Byrne TH, Kane VR, Culhane DP. Universal screening for homelessness and risk for homelessness in the Veterans Health Administration. Am J Public Health. 2013;103 Suppl 2(Suppl 2):S210-1.

13. Montgomery AE, Fargo JD, Kane V, Culhane DP. Development and validation of an instrument to assess imminent risk of homelessness among veterans. Public Health Rep. 2014;129(5):428-36.

14. Palakshappa D, Scheerer M, Semelka CT, Foley KL. Screening for Social Determinants of Health in Free and Charitable Clinics in North Carolina. J Health Care Poor Underserved. 2020;31(1):382-97.

15. Pinkerton EA, Vu M, Lindau ST. Health-related socioeconomic risk screening in outpatient obstetrics and gynecology practice. Am J Obstet Gynecol. 2022.

16. Hooshyar D, Bradshaw LD, Robinson RJ, Surís AM, LePage JP, North CS. Redesign of a Screening Process for VA Homeless Housing. Fed Pract. 2015;32(4):38-43.

17. Byhoff E, De Marchis EH, Hessler D, Fichtenberg C, Adler N, Cohen AJ, et al. Part II: A Qualitative Study of Social Risk Screening Acceptability in Patients and Caregivers. Am J Prev Med. 2019;57(6 Suppl 1):S38-S46.

18. Chhabra M, Sorrentino AE, Cusack M, Dichter ME, Montgomery AE, True G. Screening for Housing Instability: Providers' Reflections on Addressing a Social Determinant of Health. J Gen Intern Med. 2019;34(7):1213-9.

19. Morone J. An Integrative Review of Social Determinants of Health Assessment and Screening Tools Used in Pediatrics. J Pediatr Nurs. 2017;37:22-8.

20. Sokol R, Austin A, Chandler C, Byrum E, Bousquette J, Lancaster C, et al. Screening Children for Social Determinants of Health: A Systematic Review. Pediatrics. 2019;144(4).

21. Eder M, Henninger M, Durbin S, Iacocca MO, Martin A, Gottlieb LM, et al. Screening and Interventions for Social Risk Factors: Technical Brief to Support the US Preventive Services Task Force. JAMA. 2021;326(14):1416-28.

22. Montgomery AE, Rahman A, Chhabra M, Cusack MC, True JG. The Importance of Context: Linking Veteran Outpatients Screening Positive for Housing Instability with Responsive Interventions. Adm Policy Ment Health. 2021;48(1):23-35.
