# Supplementary material for: Screening for Unstable Housing in a Healthcare Setting
Source: Public Health Rev. 2023 Dec 27;44:1606438. doi: 10.3389/phrs.2023.1606438 (PMC10777743; doi:10.3389/phrs.2023.1606438)
Supplement: Supplementary file 2 [file DataSheet1.docx]

**Supplementary File 1: Search strategy**

P: "Homeless Persons"[Mesh] OR "Housing Instability"[Mesh] OR homeless* OR (("Housing"[Mesh] OR accomodat* OR home* OR hous*) AND (instabil* OR insecur*))

I: "Mass Screening"[Mesh] OR "Interviews as Topic"[Mesh] OR "Surveys and Questionnaires"[Mesh] OR screen* OR survey* OR questionn* OR interview*

C: N/A

O: N/A

NOT "Food"[Mesh] OR food

Last ten years

OR

"Social Determinants of Health"[Mesh] OR "Socioeconomic Factors"[Mesh] OR socioeconomic OR "social determinants" OR "social determinant"

AND

"Risk Assessment"[Mesh] OR "Risk"[Mesh] OR risk

AND

"Mass Screening"[Majr] OR screen*[title]

Google Scholar:

Screening for homelessness
